# Prediction of Pullout Behavior of Belled Piles through Various Machine Learning Modelling Techniques

**DOI:** 10.3390/s19173678

**Published:** 2019-08-24

**Authors:** Dieu Tien Bui, Hossein Moayedi, Mu’azu Mohammed Abdullahi, Ahmad Safuan A Rashid, Hoang Nguyen

**Affiliations:** 1 Institute of Research and Development, Duy Tan University, Da Nang 550000, Vietnam; 2 Geographic Information System Group, Department of Business and IT, University of South-Eastern Norway, N-3800 Bø i Telemark, Norway; 3 Department for Management of Science and Technology Development, Ton Duc Thang University, Ho Chi Minh City, Vietnam; 4 Faculty of Civil Engineering, Ton Duc Thang University, Ho Chi Minh City, Vietnam; 5 Civil Engineering Department, College of Engineering, University of Hafr Al-Batin, Al-Jamiah 39524, Eastern Province, Kingdom of Saudi Arabia; 6 Center of Tropical Geoengineering (Geotropik), School of Civil Engineering, Faculty of Engineering, Universiti Teknologi Malaysia, Johor Bahru 81300, Malaysia; 7 Department of Surface Mining, Hanoi University of Mining land Geology, 18 Vien Street, Duc Thang Ward, Bac Tu Liem District, Hanoi, Vietnam; 8 Center for Mining, Electro-Mechanical Research, Hanoi University of Mining and Geology, 18 Vien Street, Duc Thang Ward, Bac Tu Liem District, Hanoi, Vietnam

**Keywords:** machine learning, belled piles, pullout behavior

## Abstract

The main goal of this study is to estimate the pullout forces by developing various modelling technique like feedforward neural network (FFNN), radial basis functions neural networks (RBNN), general regression neural network (GRNN) and adaptive neuro-fuzzy inference system (ANFIS). A hybrid learning algorithm, including a back-propagation and least square estimation, is utilized to train ANFIS in MATLAB (software). Accordingly, 432 samples have been applied, through which 300 samples have been considered as training dataset with 132 ones for testing dataset. All results have been analyzed by ANFIS, in which the reliability has been confirmed through the comparing of the results. Consequently, regarding FFNN, RBNN, GRNN, and ANFIS, statistical indexes of coefficient of determination (*R^2^*), variance account for (VAF) and root mean square error (RMSE) in the values of (0.957, 0.968, 0.939, 0.902, 0.998), (95.677, 96.814, 93.884, 90.131, 97.442) and (2.176, 1.608, 3.001, 4.39, 0.058) have been achieved for training datasets and the values of (0.951, 0.913, 0.729, 0.685 and 0.995), (95.04, 91.13, 72.745, 66.228, 96.247) and (2.433, 4.032, 8.005, 10.188 and 1.252) are for testing datasets indicating a satisfied reliability of ANFIS in estimating of pullout behavior of belled piles.

## 1. Introduction

Belled piles constructed from concrete and have been designed to raise the bearing capacity of embedded piles. On the other hand, the base geometry is as inverted to the cone. Accordingly, different computational models have been used to analyze the pile behavior in various independent loadings, lateral loadings, vertical-uplift, and vertical compressive [1,2,3,4,5], besides, the forecasting of the (1) bearing capacity of pile foundation [6,7]; (2) uplift capacity of suction caisson [8]; (3) pile dynamic capacity [9,10]; (4) pile setup [11]; and (5) pile settlements [12] has defined artificial neural network (ANN) to forecast the pullout capacity of suction foundations through the applying of a database, including the results of centrifuge tests. Moreover, Ardalan et al. [13] have investigated GMDH (group method of data handling from neural networks’ family) with GA (genetic algorithms) indicating the effectual cone point resistance and cone sleeve friction on the inputs values of pile unit shaft resistance. Furthermore, Alavi et al. [14] have explained TGP (tree formed genetic programming), LGP (linear-genetic programming) and GEP (gene expression programming) to surpass the prediction equation formula of the pullout capacity of suction caissons utilizing dataset according to literature. ANFIS in geotechnical engineering is well explained by Cabalar et al. [15]. Yilmaz et al. [16] have studied artificial neural networks (ANNs) and ANFIS to predict the permeability of coarse-grained soils. Cheng et al. [17] have used RBF (radial basis function) neural network hybrid inference model (IFRIM), artificial bee colony (ABC), and fuzzy logic (FL) to forecast the suction caissons’ pullout capacity. Wu et al. [18] have defined an analysis method in a single axially loaded bored pile through the conduction of a nonlinear soft method resulting that the skin friction’s soft features in the bored pile have not been appropriately simulated when the pile has been adjusted to small loads. Thomas et al. [19] have developed an innovative model as randomized ANFIS to forecast the ground motion’s parameters associated with seismic signals. Ganjidoost et al. [20] have used ANFIS and Genetic Algorithms (GAs) (joint applying) to forecast the soil permeability coefficient. The potential of ANFIS to say resilient modulus of flexible pavements subgrade soils has also been investigated by Sadrossadat et al. [21]. On the other hand, Shi et al. [22] has presented a few field tests on bearing capacity of enlarged base piles, besides the verifying of the primary majors affecting the load and deformation behaviors of enlarged piles, such as installing method, main size of piles and hydro-geologic case resulting that the belled piles in length (5 m and 15 m) are grouped as the end bearing piles, due to the lack of skin friction design in a pile bearing capacity.

Chae et al. [23] have studied the pullout resistance of belled pile located in weathered sandstones of Persian Gulf, including few full-scale pullout loading experiments on piles’ belled tension located in Abu Dhabi. The comparison of 3D finite element (FE) results and theoretical models, the later has overestimated the final pullout resisting of the belled pile without the bell-shaped consideration [23]. Xu and Hou [24] have investigated the belled large-diameter PHC pipe’s behavior resulting that the ultimate bearing resistance of that pipe is superior to the regular bored pile. Zhu et al. [25] have investigated the uplift resistance of a new type umbrella-shaped ground anchor in clay along with the tests (field and laboratory uplift tests) and numerical perspective. Therefore, the outcome has revealed that conventional anchors have lower uplift capacity than the new type of umbrella-shaped anchor. The anchor embedment depth and diameter have influenced the anchors uplift resistance. An elastic-plastic computing solution for the pullout of the belled pile has been performed by Yao and Chen [26]. Qian et al. [27] were examined the behavior of 15 belled shafts for many collapsible losses used into loess soils, in an arid environment, and 18 tensile uplifts straight side. The considered environments for these tests were documented. After that, belled shafts into the loess and tensile uplift straight-sided were suggested to modify regarding the capacity and also displacement. Scholars developed diverse methods for predicting the pile’s pullout capacity like ABC algorithm, RBF (IFRIM), and FL recently [28,29], but there are not uniform and also reliable results (under different conditions) that making uncertainty in predicting the pullout capacity. In addition, there is not a proper analysis according to an extensive number of experimental laboratory schedule. It should be noted that only a few pieces of research are performed in belled piles, because of problems existing in large-scale laboratory data collection and full scale.

Through the use of ANFIS, this study has performed a reliable prediction in the belled pile’s pullout capacity of embedded in coarse-grained soil. Therefore, the angle of enlarged base, base diameter, embedment ratio, and shaft diameter have been taken as input, while the belled pile’s pullout capacity is output. This article consists of: (i) The introduction providing the literature with the significance of the study; (ii) methodology that explains four of the proposed machine learning-based solutions; (iii) data collection and model evaluation that defined the training and testing datasets subjected to various machine learning methods. The model evaluation section also exhibits the obtained results and provide an initial comparison between the predicted outputs from each of the four proposed techniques; and finally (iv) network performance of the fuzzy system (i.e., selected as the best machine learning solution for this study) provided the comparison charts of the actual and ANFIS-based predicted *Pu*.

## 2. Methodology

### 2.1. Machine Learning Techniques

Several machine learning techniques have been used to forecast the final pullout capacity of the under-reamed piles placed in dense and loose coarse-grained soil.

#### 2.1.1. Adaptive Neuro-Fuzzy Inference System (ANFIS)

**Figure 1:**
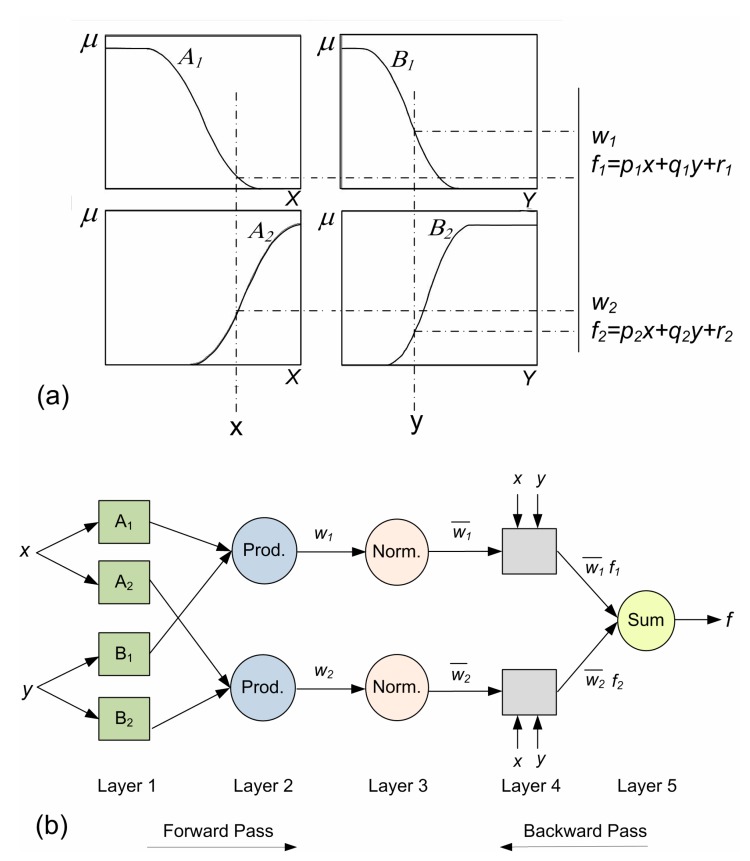
(**a**) Sugeno model mechanism; (**b**) its equivalent adaptive neuro-fuzzy inference system (ANFIS) structure.

Fuzzy inference system is to say any real continuous function in a complex set by using ANNs [30] in which the input has been planned to the input membership parameters mapped out to a set of fuzzy *if-then* rule mapped out to output parameters planed to the output membership functions that are mapped out to a single output. Regarding Fuzzy inference system (FIS) with one output (*f*) and inputs (*x, y*), an individual fuzzy *if-then* rule in the case of the first-order Sugeno model (Figure 1a,b) is:

Rule 1: *If x is A*_1_ *and y is B*_1_*,* 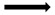 *f*_1_ = *p*_1_*x* + *q*_1_*y* + *r*_1_
Rule 2: *If x is A*_2_ *and y is B*_2_*,* 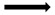 *f*_2_ = *p*_2_*x* + *q*_2_*y* + *r*_2_

*p_i_*, *q_i_* and *r_i_ (i =* 1,2*)* are the output linear factors (consequent parameters). ANFIS with five layers has been explained as follows (Figure 1b):

**Layer 1:** All adaptive nodes of the layer has included one node as Equations (1) and (2):

(1) $$O_{1,i}=\mu_{A_{i}}(x),i=1,2,$$

(2) $$O_{1,i}=\mu_{B_{i-2}}(y),i=3,4,$$

*i* stands for the membership grade for a set of fuzzy (A_1_, A_2_, B_1_, B_2_).

*O*_1,*i*_ shows the node output *i* in layer 1.

Gaussian function as a common membership function in Equation (3):

(3) $$\mu_{A}\left(x\right)=\exp\left(-\frac{{\left(x-c\right)}^{2}}{2\sigma^{2}}\right).$$

In Equation (6), *c* and σ are the premise parameters.

**Layer 2:** The certain nodes of the layer multiply all input signals representing the firing intensity of order as Equation (4):

(4) $$O_{2,i}=w_{i}=\mu_{A_{i}}\left(x\right)\mu_{B_{i}}\left(y\right),\;\;i=1,2.$$

**Layer 3:** In the layer, the fixed nodes has computed the ratio of *i*-th rule’s firing strength for the summation of whole rule’s firing strengths called the normalized firing strength as Equation (5):

(5) $$O_{3,i}=\bar{w_{i}}=\frac{w_{i}}{w_{1}+w_{2}},i=1,2$$

**Layer 4:** The adaptive nodes have the node functions as Equation (6):

(6) $$O_{4,i}=\bar{w_{i}}f_{i}=\bar{w_{i}}\left(p_{i}x+q_{i}y+r_{i}\right),\;\;\;i=1,2.$$

$\bar{w_{i}}$ shows a normalized firing strength of layer 3.

$\left\{p_{i},q_{i},r_{i}\right\}$ stand consequent parameters.

**Layer 5:** One fixed node of the layer labelled ∑, whole computing output as the summation of all incoming signals as Equation (7):

(7) $$O_{5,i}=\underset{i}{\sum }\bar{w_{i}}f_{i}=\frac{\sum_{i}w_{i}f_{i}}{\sum_{i}w_{i}},\;i=1,2.$$

**Figure 2:**
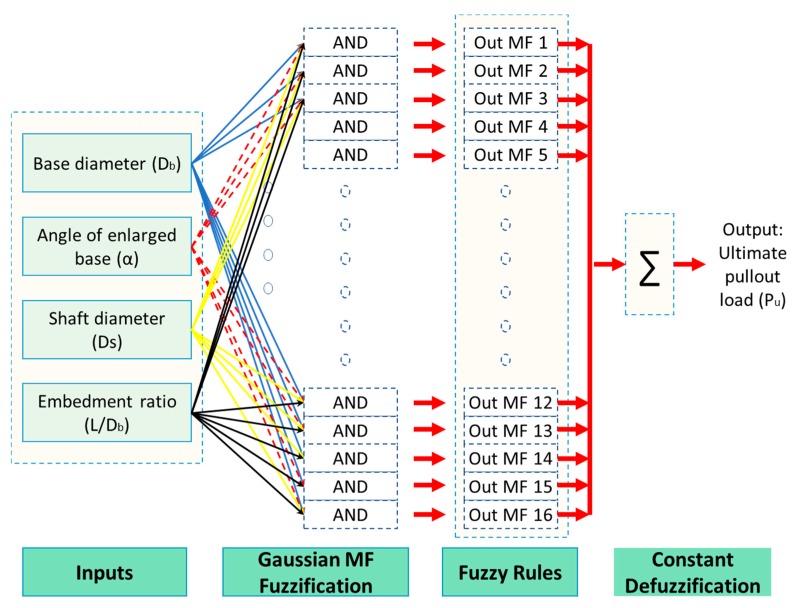
The schematic view of the ANFIS model.

ANFIS has included two parameter sets as (1) adaptive and (2) consequent defined by using a two-step process as a hybrid algorithm. While the former (adaptive) is used to be constant, the later (consequent) is computed by the least-squares method called forward pass. In the backward pass (second process), the ultimate factors are constant, while the adaptive is gained by gradient descends method. On obtaining the model parameters, output values are computed for all order-paired of training data, then compared to the model’s anticipated values (see more in seeing Jang and Sun [30] and Cabalar et al. [15]). In the case of the appropriate performance of the model, the gap between the predicted and observed data has gained the lowest error ratio. Meanwhile, in this paper, ANFIS has been applied to forecast ultimate under-reamed piles’ pullout capacity embedded in dense and loose coarse-grained soil, so the input parameters have been obtained as; (1) base diameter (*D_b_*), (2) angle of enlarged base (α), (3) shaft diameter (*D_s_*), (4) embedment ratio (*L/D_b_*) and (5) ultimate pullout load (*P_u_*) (output parameter). ANFIS data set (Table 1) has been divided into two datasets as (1) training and (2) testing, in which MATLAB 7.0.4 has been applied to train ANFIS indicating the characteristic of the most appropriate obtained ANFIS model (Figure 2).

#### 2.1.2. Feedforward Neural Network (FFNN)

**Figure 3:**
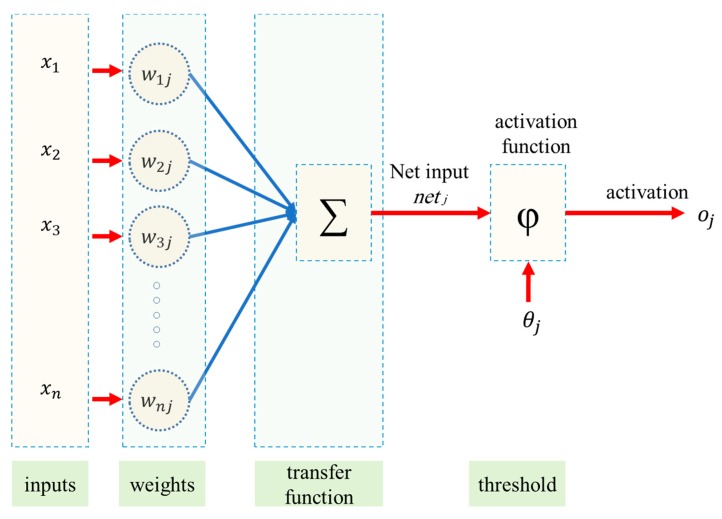
A view of the feedforward neural network (FFNN) approach.

FFNN, as a simple structure, has been applied for (1) modelling many non-linear phenomena [31,32,33] (2) forecasting results for complex systems comprising (a) one layer of input, (b) one output layer, and (c) one or more hidden layer(s). The input consists of pre-mapped in the layer of input prior to enter the hidden layer, accordingly. In hidden layers and also output layer, the information multiplied with a weight matrix that is joined to a bias vector and transferred, so add block (+) has represented a plain operator of vector summation; On the other hand, for hidden layers, the transfer functions are any tangent sigmoid-like logarithm sigmoid. Figure 3 has shown a single-layer FFNN. The transfer function of the hidden layer (*f_1_*), as well as the output layer (*f_2_*), has been regarded to be pure linear (*purelin*) and tangent sigmoid (*tansig*). While the *purely* function of variable *x* has returned the identical value of *x*, the function of *tansig* for *x* has returned an amount of (−1 and 1) as Formulas (8) and (9):

(8) purelin(x)=x,

(9) $$tansig\left(x\right)=\frac{2}{1+e^{-2x}}-1.$$

#### 2.1.3. Radial Basis Function Based Neural Network (RBNN)

**Figure 4:**
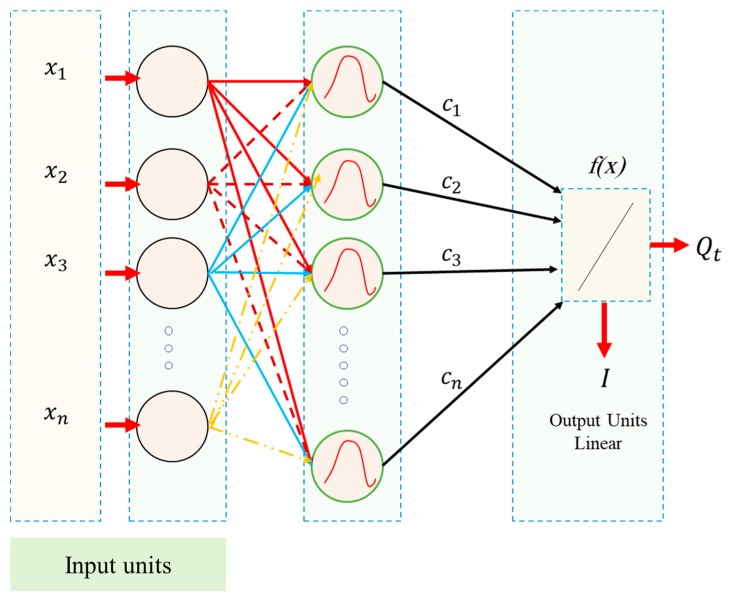
The schematic view of the radial basis functions neural networks (RBNN) structure.

Broomhead and Lowe [34] have suggested the RBNN for the neural network. Figure 4 shows the RBNN layers. As seen, there are two different layers, which output nodes make a linear set of the basis functions. When the input added into a small localized area of the input space, these functions that are in the hidden layer generate a considerable non-zero response to the only input stimulus. In Ref. [35], this approach has been introduced as a localized, acceptable field network [35]. Figure 2 shows the relation between input and output. As seen, in empirical modelling, the input transformation is necessary for fighting the curse of dimensionality. With a radial constant shape basis function, the input transformation type of the RBNN is the local nonlinear paradigm. The radial base functions have an effective role as regressors, since squashing the multi-dimensional input without applying any the output space, nonlinearly. After a linear regressor is considered in the output layer, the adjustable factor is the regressor weight. By utilizing the linear least square method, these factors can be easily determined. Moreover, it suggests a proper advantage in the case of convergence. We have described an algorithm of the RBNN and the basic concept as follows:

We have introduced a nonlinear function *h(x,t)* (*x* stand for the input variable, and also *t* shows its center) that is named a radial basis function. It is based on the radial distance r=∥x−t∥. *N* is real numbers $\left\{y_{i}\in R|i=1,2,3,\ldots ,N\right\},$ and *N* stands for different points $\left\{x_{i}\in \;R^{n}|i=1,2,3,\ldots ,N\right\}$, we can obtain the function (*f*) from $R^{n}$ to *R* satisfying the interpolation states: $f\left(x_{i}\right)=y_{i},\;i=1,2,3,\ldots ,N$. The approach of RBNN includes in selecting the function a linear space of dimension *N* that is based on the data points $\left\{x_{i}\right\}$. This space was selected as a set of functions.

(10) $$\left\{h\left(∥x-x_{i}∥\right)|i=1,2,3,\ldots ,N\right\}.$$

∥ shows the Euclidean norm of $R^{n}$. Hence, the solution of the interpolation issue may be obtained as the below form:

(11) $$f\left(x\right)=\overset{N}{\underset{i=1}{\sum }}c_{i}h\left(∥x-x_{i}∥\right).$$

One can obtain the unknown parameters ($c_{i})$ setting the interpolation states $f\left(x\right)=y_{i},\;\left(i=1,2,3,\ldots ,N\right)$ on Equation (12). It can yield the linear system.

(12) $$f\left(x\right)=\sum_{i=1}^{N}c_{i}h\left(∥x_{j}-x_{i}∥\right)\;,\;j=1,2,3,\ldots ,N.$$

The coefficients $c_{i}$ can be determined by $c=H^{-1}y$ and considering the vectors *y*, *c* and the symmetric matrix *H* as ${\left(y\right)}_{j}-y_{j},\;{\left(c\right)}_{i}-c_{i},$ and $\left(H_{ij}\right)-\;h\left(∥x_{j}-x_{i}∥\right)$. Therefore, the RBNN is a particular case of a linear regression approach. The RBNN approach carries out linear regulation of the weights in the case of radial bases and does not do factor learning as in the back-propagation networks. This specific of the RBNN provides the benefit of a quick converging time without local minima. In the present work, we have exanimated many numbers of hidden layer neurons. After that spread constants were tested for the models of RBNN with a plain trial and error approach may add some loops in the program codes.

In general, similar to the FFNN, RBNN technique is a single layer (as shown in Figure 4) indicating the input and output of the network, meanwhile, in the hidden layer, a radial basis (*radbas*) features has been used (instead of a sigmoid function) adding that radial basis was a transferring action taking *x* variance and providing the value of (0–1) as formulating in Equation (13):

(13) $$radbas\left(x\right)=\exp\left(-x^{2}\right).$$

Additionally, in the hidden layer, an element - element vector operator has been applied instead of a vector adding (+) operator known as dot product operator (*) containing many neurons, due to their simple designing and training. In the availability of the large quantity of training data, RBNN is preferable. Moreover, RBNN has been conducted through the neurons addition. Because quantity equality exists among the neurons along with the vectors of input data.

#### 2.1.4. Generalized Regression Neural Networks (GRNN)

**Figure 5:**
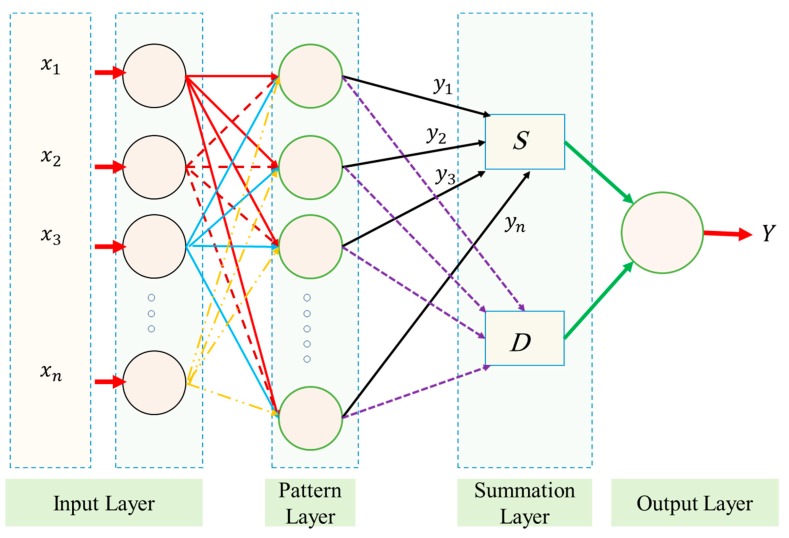
A general regression neural network (GRNN) diagram.

Figure 5 shows a GRNN schematic. In the Ref. [20] the theory of the GRNN has been introduced and stated that GRNN has four layers: Summation, input, layers of output, and pattern. In the first layer, the total number of factors are identical to the number of input units. In addition, this layer is connected to the pattern layer that is known as the second layer. In this layer, each unit indicates a training pattern, and also its output can be a measure of the distance of input from the stored patterns. In the layer of summation, each unit of pattern layer has been connected to the two neurons as follows:

S-summation neuron as well as D-summation neuron.

D-summation neuron computes the unweighted outputs in the case of the pattern neurons. In addition, the S-summation neuron also calculates the weighted outputs summations of the pattern layer. In the layer of pattern, we can show the connection of weight among the *i_th_* neuron and the S-summation neuron by $y_{i}$ and unity is the connection weight in the case of D-summation neuron. The layer in output distributes the output of each S-summation neuron, merely, using that of each neuron in D-summation, leading the estimated value to an undetermined input vector *x* as

(14) $$y_{i}\left(x\right){=}_{\overset{n}{\underset{i=1}{\sum }}Exp\left[-D(x-x_{i}\right]}^{\overset{n}{\underset{i=1}{\sum }}y_{i}Exp\left[-D(x-x_{i}\right]}.$$

*n* indicates the number of training patterns, and the Gaussian *D* function in Equation (15) is defined as

(15) $$D\left(x,x_{i}\right)=\overset{p}{\underset{j=1}{\sum }}{\left(\frac{x_{j}-x_{ij}}{\zeta }\right)}^{2}.$$

*p* stands for the number of parameters in a vector of input. The $x_{j}$ and $x_{ij}$ show the *j_th_* element of *x* and $x_{i}$, respectively. The ζ relates to the spread factor, which the optimized value can be experimentally determined [36]. In this way, a large spread relates to a function of smooth approximation. Too large a spread is defined many neurons that need to appropriate a fast-changing function. Moreover, it also causes the network to malfunction. In the present work, various spreads have been exanimated for discovering the best value in the case of the given issue. Noted that, as for the method of backpropagation, GRNN does not require an iterative training trend [37].

### 2.2. Data Collection

**Figure 6:**
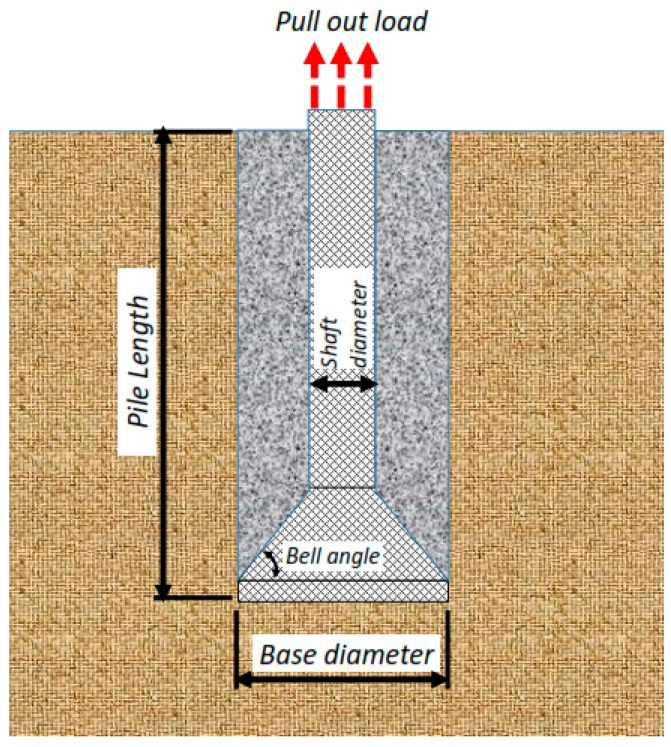
A sample of the enlarged base pile and key parameters for modelling.

**Figure 7:**
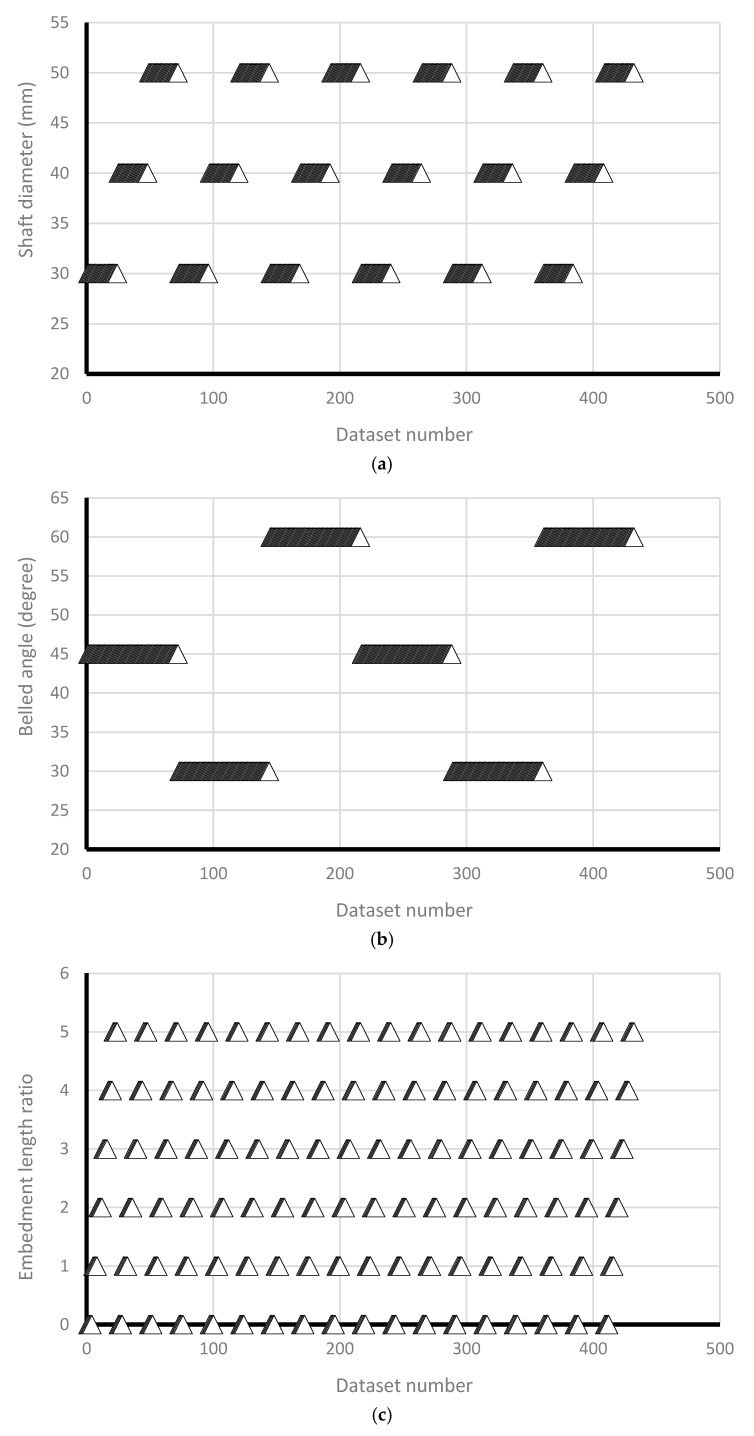

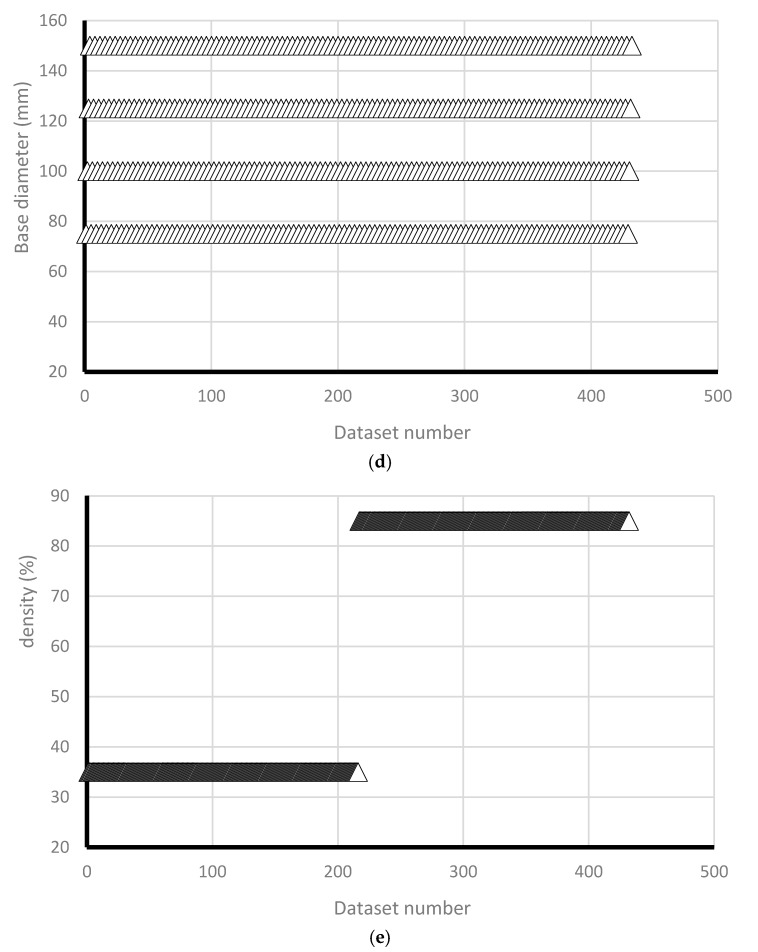
The graphical database used (i.e., training and testing) versus dataset number; (**a**) Shaft diameter (mm); (**b**) Belled angle (degree); (**c**) Embedment length ratio; (**d**) Base diameter (mm); (**e**) density (%).

In the current study, few data sets defined by Nazir et al. [38] have been used based on a series of small scale laboratory tests. Considering material parameters used in ANFIS and laboratory work, the input parameters have included (1) enlarged base angle (α) of 30, 45, and 60 degree, (2) shaft diameter (D_s_) of 30, 40, and 50 mm, (3) base diameter (D_b_) of 75, 100, 125, and 150 mm, and four embedment ratio (L/D_b_) of 1, 2, 3, 4, and 5, while the output parameter is pullout load (P_u_) (Figure 6). The uplift resistance (i.e., obtained from a series of experimental work) collected from a laboratory works presented by Nazir et al. [38] is shown (Table 2). Figure 7 shows the graphical database utilized that means training and testing).

## 3. Results and Discussion

### 3.1. Model Evaluation

Considering the results of pullout behavior forecasting of belled piles, R^2^, RMSE and variance account for (VAF) have been used providing the equations as follows:

(16) $$R^{2}=1-\;\frac{\sum_{i=1}^{N}{\left(y-\;y\prime \right)}^{2}}{\sum_{i=1}^{N}\;{\left(y-\tilde{y}\right)}^{2}},$$

(17) $$\mathrm{VAF}=[1-\;\frac{\mathrm{var}(y-\;y\prime )}{\mathrm{var}(y)}]\times 100,$$

(18) $$\mathrm{RMSE}\;=\;\sqrt{\frac{1}{N}\overset{N}{\underset{i=1}{\sum }}\;{\left(y-\;y\prime \right)}^{2}}.$$

y and y′ as forecasted and measured variances.

$\tilde{y}$ as the mean of y and N as whole data.

The method has been performed superlatively if R^2^ = 1, VAF = 100 and RMSE = 0. Comparing the aforementioned methods (ANFIS, FFNN, RBNN, and GRNN) with the results achieved by ANFIS have indicated a close performance to one another (Table 3) (see Wang [39]). In order to distinguish a supreme model, one ranking technique proposed by Zorlu et al. [40] (also well discussed in Moayedi and Hayati [41], Moayedi et al. [42] and Moayedi and Rezaei [43]) has been applied in which each performance index (VAF, RMSE, or R^2^) has been ordered in their level, so the most significant function index has shown the highest rating [44].

### 3.2. Performance of the Selected Model

**Figure 8:**
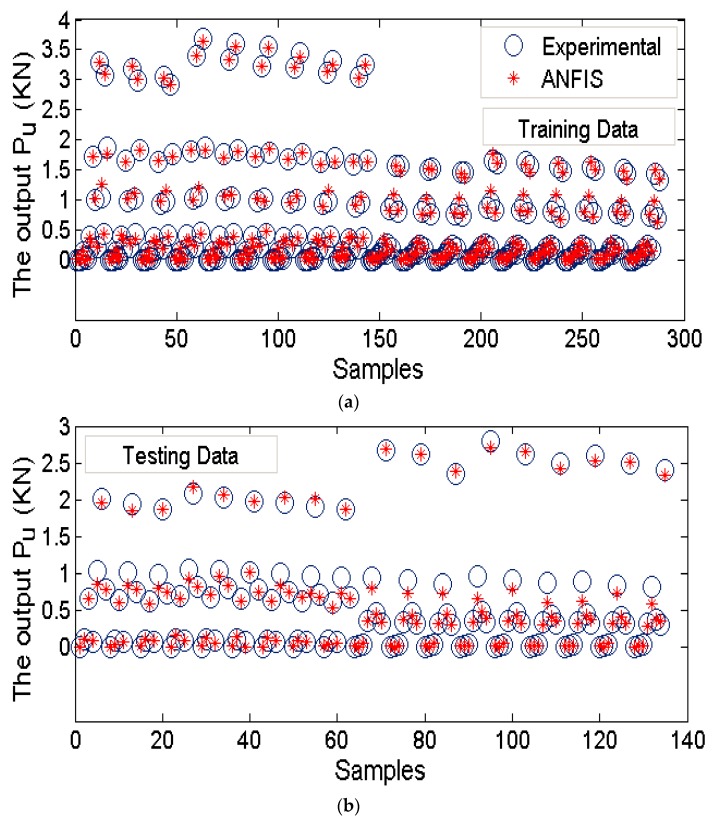
ANFIS training and testing results for *P_ult_*. (**a**) the training data, (**b**) the testing data.

**Figure 9:**
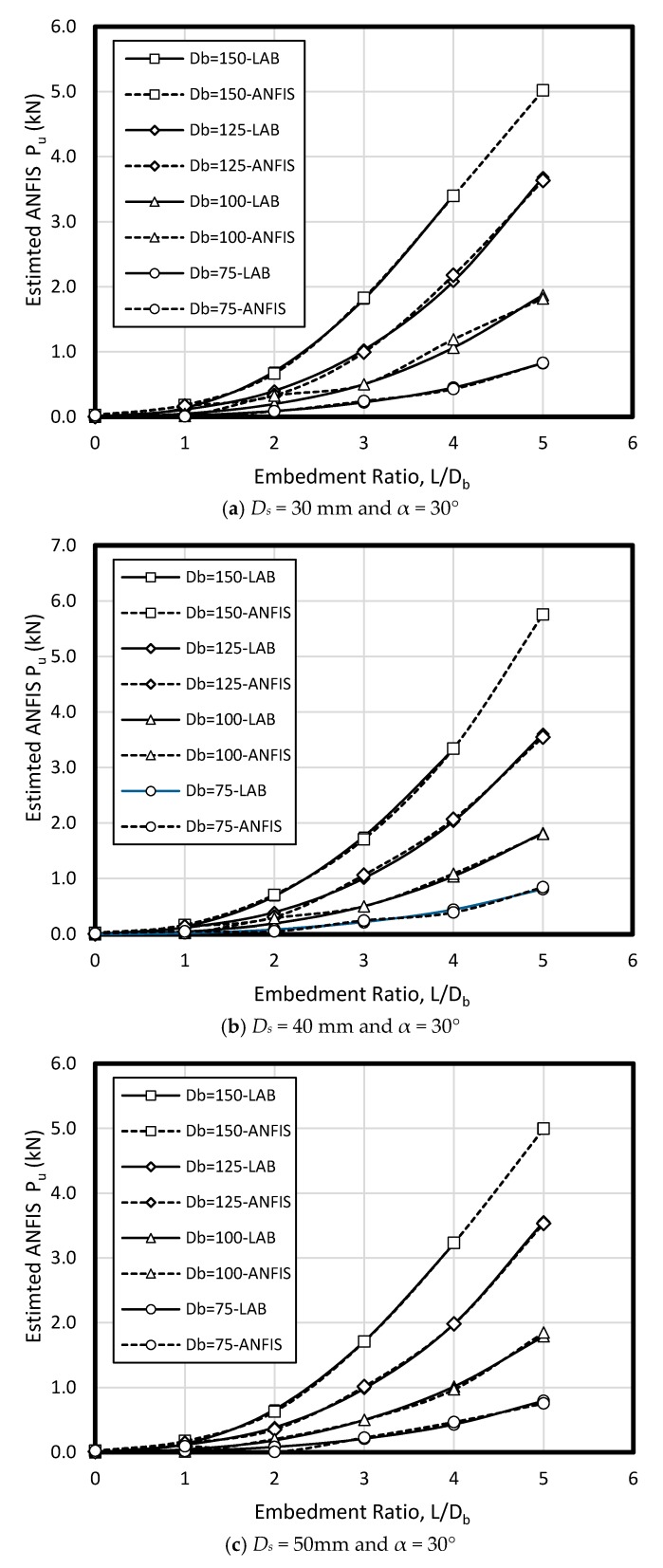
*P_u_* for various *D_s_*, *D_b_*, and *L/D_b_* in laboratory model and predicted from ANFIS when α = 30° in dense sand.

**Figure 10:**
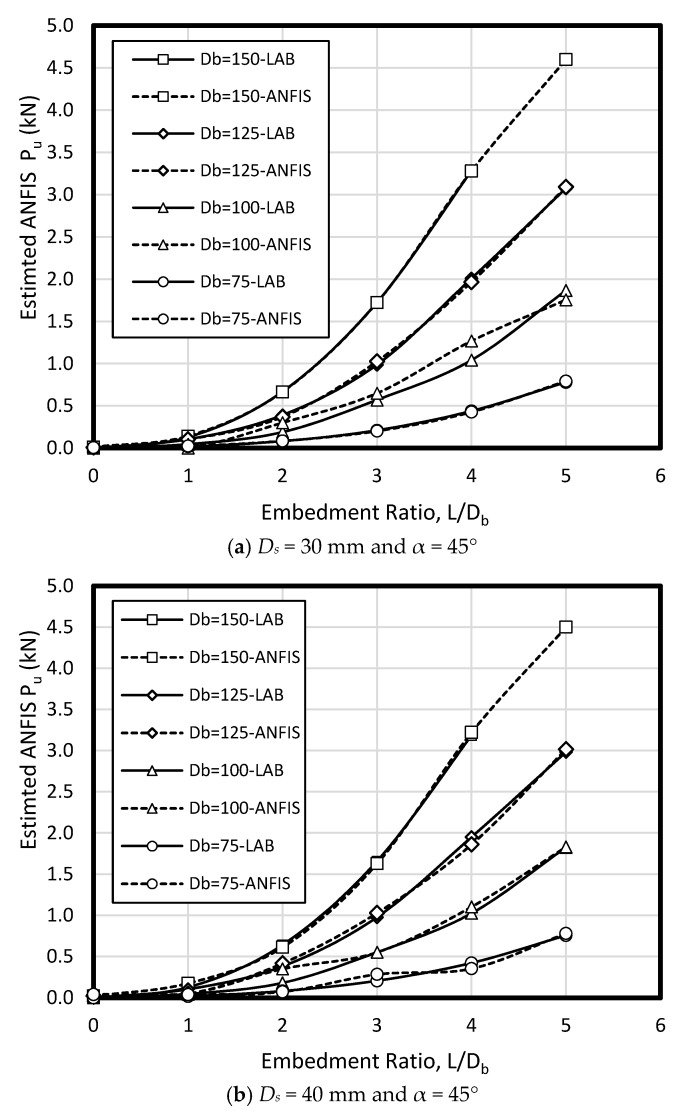

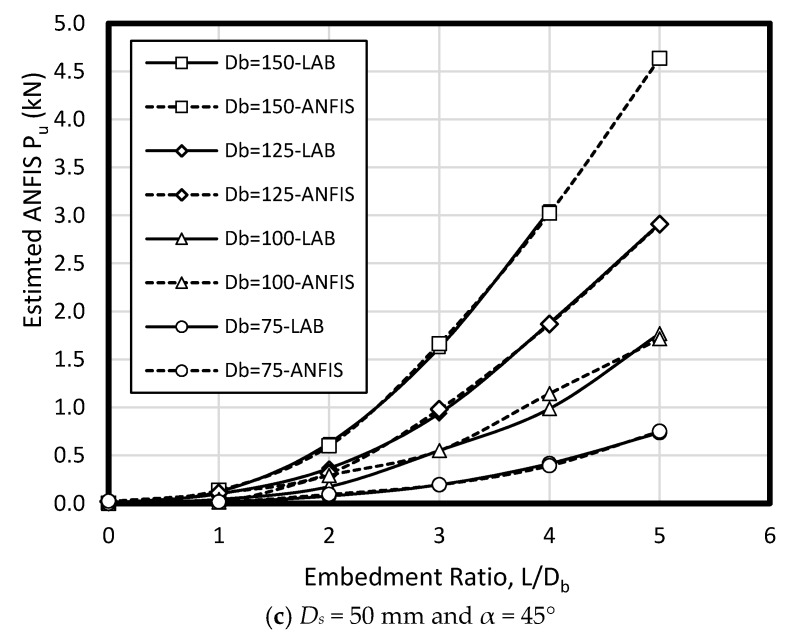
*P_u_* for various *D_s_*, *D_b_*, and *L/D_b_* in laboratory model and predicted from ANFIS when α = 45° in dense sand.

**Figure 11:**
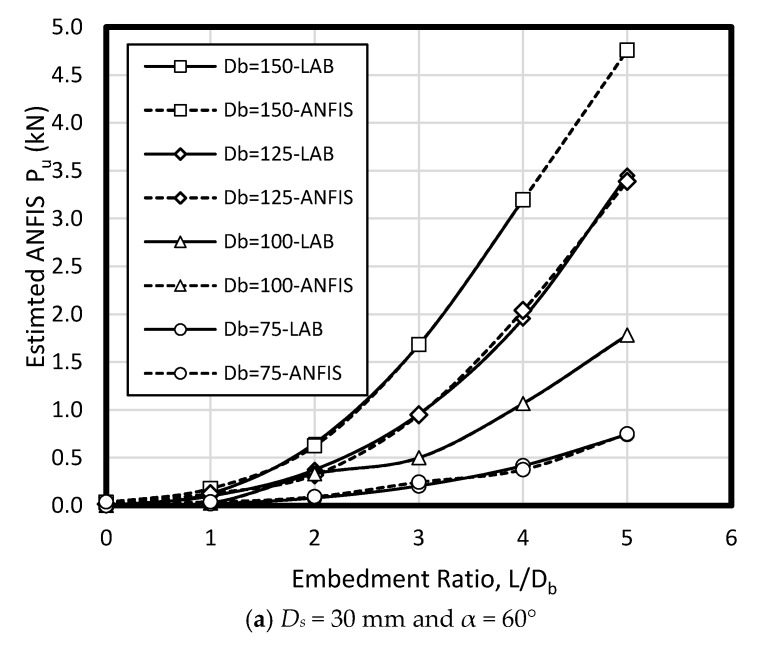

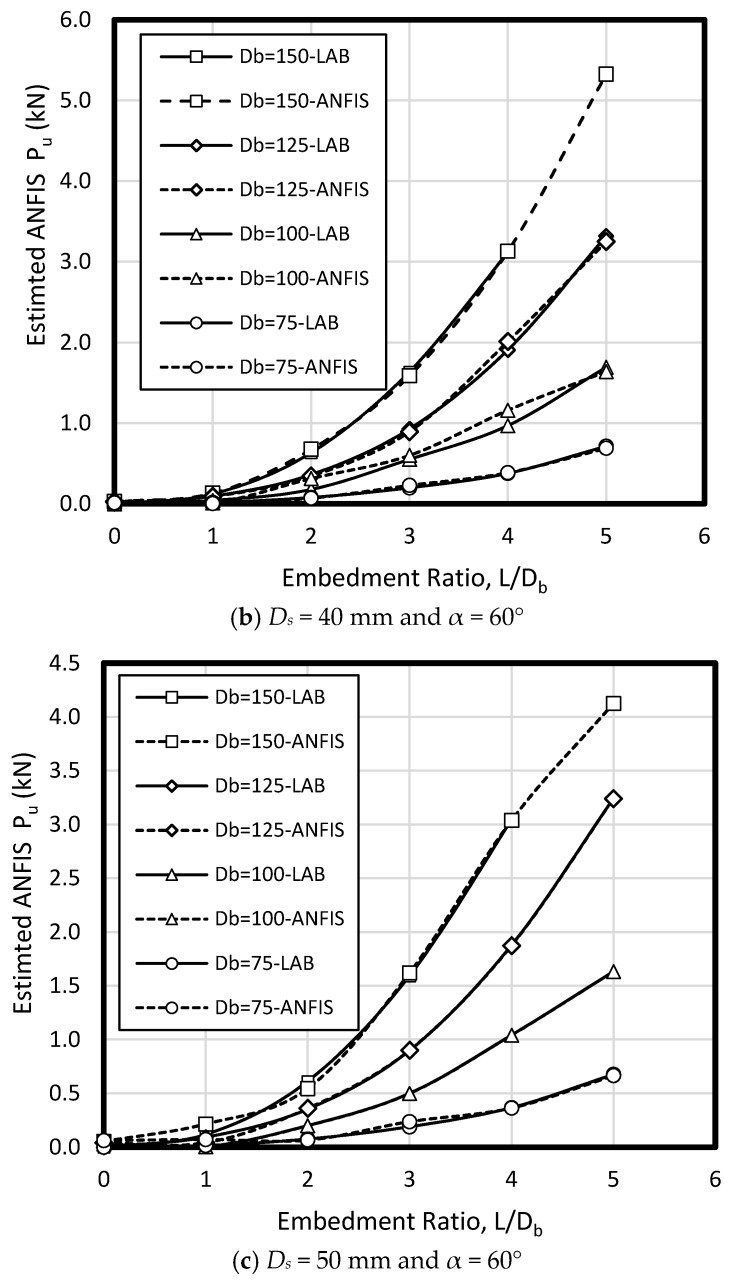
*P_u_* for various *D_s_, D_b_*, and *L/D_b_* in laboratory model and predicted from ANFIS when α = 60° in dense sand.

**Figure 12:**
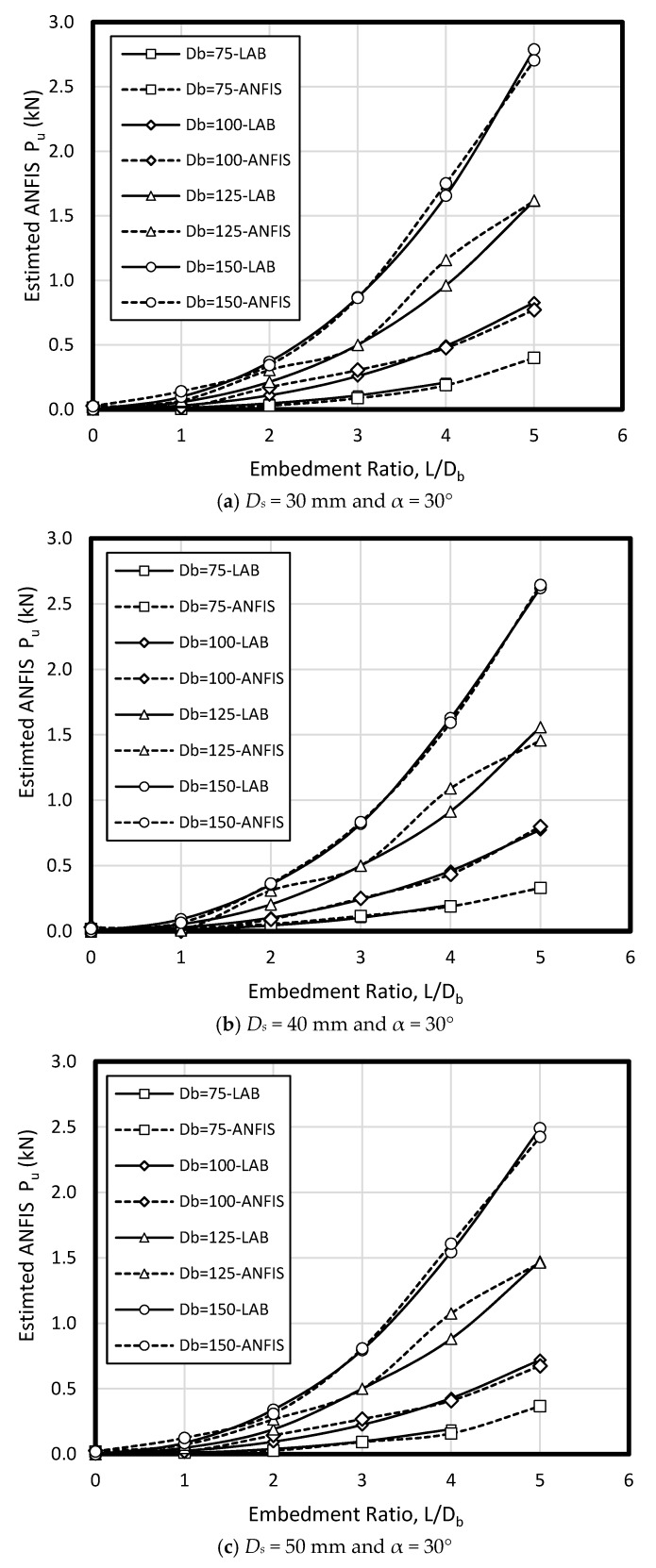
*P_u_* for various *D_s_, D_b_*, and *L/D_b_* in laboratory model and predicted from ANFIS when α = 30° in loose sand.

**Figure 13:**
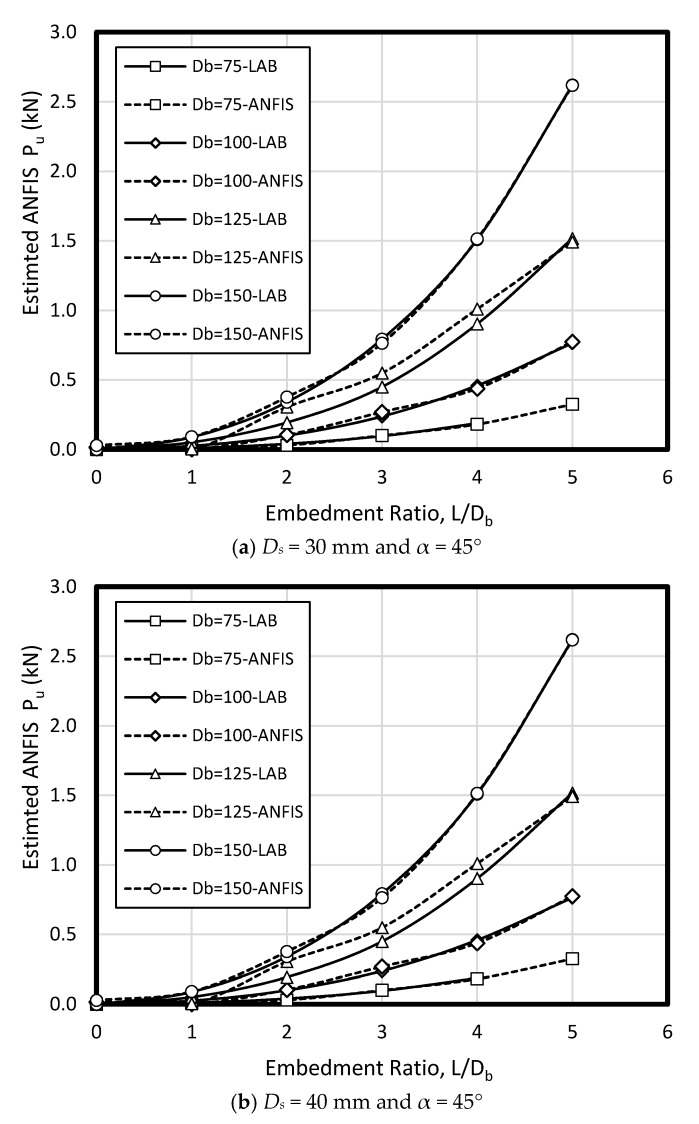

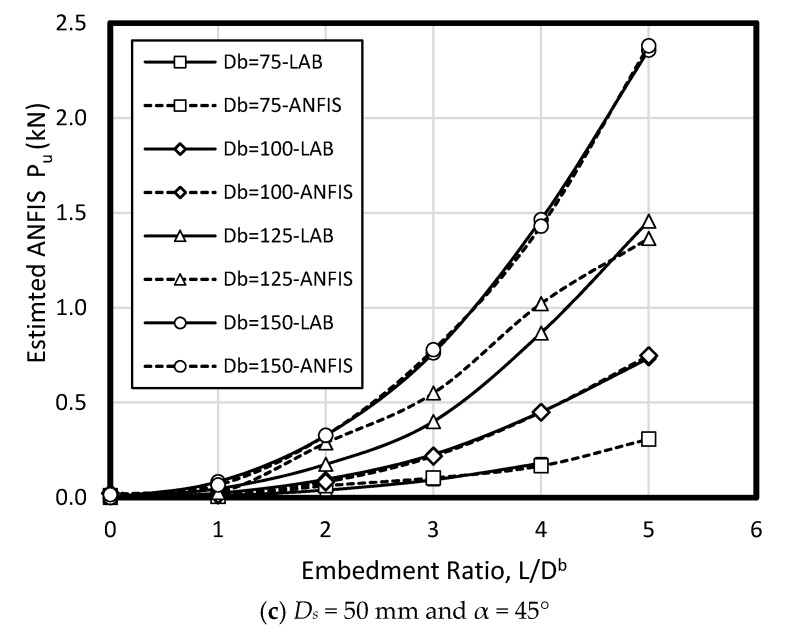
*P_u_* for various *D_s_, D_b_*, and *L/D_b_* in laboratory model and predicted from ANFIS when α = 45° in loose sand.

**Figure 14:**
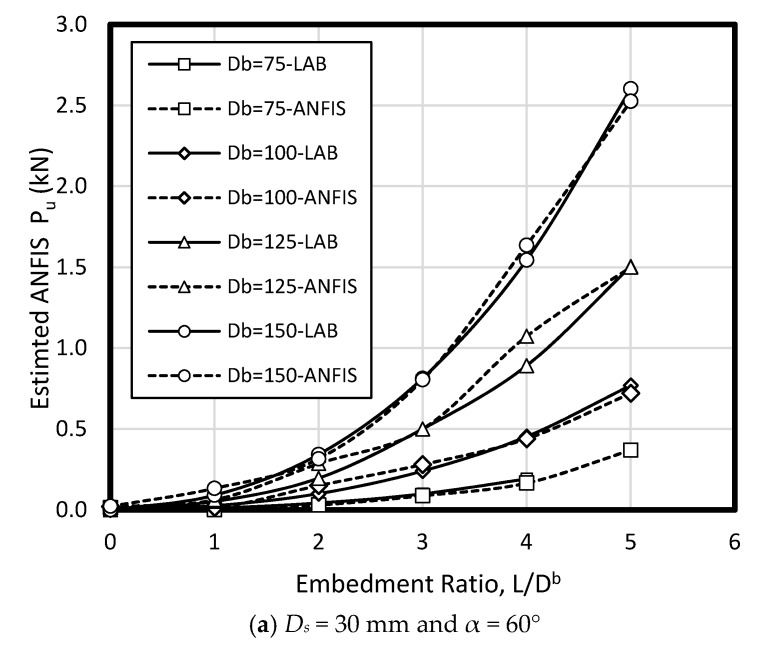

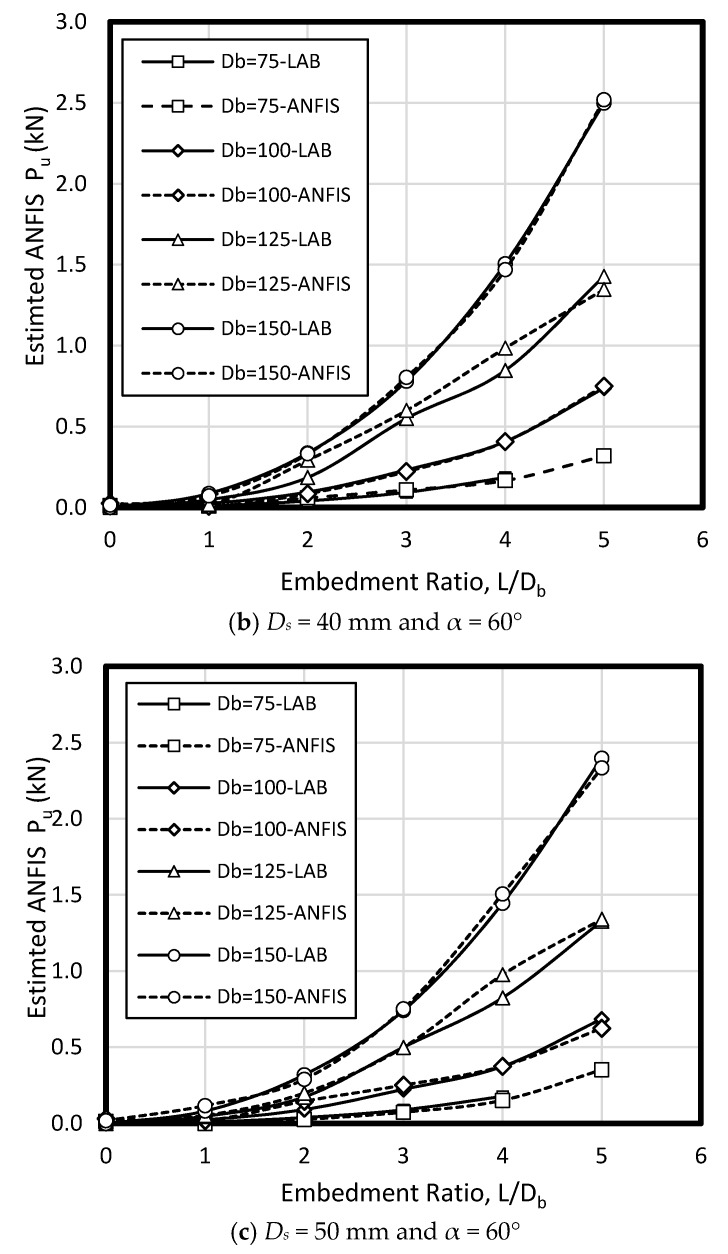
*P_u_* for various *D_s_, D_b_*, and *L/D_b_* in laboratory model and predicted from ANFIS when α = 60° in loose sand.

To verify ANFIS performance, the forecasting results have been compared to the laboratory test results. Provided data points was included of 432 samples, while 300 belonged to training dataset and 132 belonged to testing dataset (Figure 8). Accordingly, the estimated values using ANFIS is near the measured outcomes proving ANFIS as a precise and valid prediction model for ultimate upload resistance in a belled pile. The variation of the pullout capacity (*P_u_*) of the single belled pile has been presented in Figure 9, Figure 10, Figure 11, Figure 12, Figure 13 and Figure 14. The predicted values of *P_u_* obtained by ANFIS for different *D_s_*, *D_b_*, and *L/D_b_* have been compared to measure *P_u_* within the data set provides the results of *P_u_* for the belled pile embedded in dense sand (Figure 9, Figure 10 and Figure 11) and loose sand (Figure 12, Figure 13 and Figure 14). When the test results are shown with a continuous line, ANFIS results are dashed. The pullout capacity tests have been performed in various terms, as defined in Table 1. *P_u_* of the belled pile has been inclined to compare to the normal system of the simply bored pile. Considering ANFIS and LAB results, the pullout capacity in piles embedded in dense sand is higher than belled piles in loose sand, say in dense and loose sand, *P_u_* for a particular embedment ratio *L/D_b_* = 1, 2, 3, 4, 5; *D_s_* = 30 mm, α = 30°, and for *D_b_* of 150 mm are 0.03, 0.18, 0.66, 1.83, 3.40, 5.02 kN, and 0.03, 0.14, 0.34, 0.86, 1.75, 2.70 kN (refer to Figure 9a, Figure 10a, and Figure 11a). The pullout capacity has been raised on soil density increment, base diameter enlargement, embedding ratio and slightly decline with the shaft diameter confirmed by the value of *P_u_* obtained from ANFIS. For example, to control the influence of the bell’s diameter on the pullout behavior of belled pile from the proposed model, the pullout pile load in dense soil condition are 0.79, 1.76, 3.09, and 4.60 kN when for the loose sand with a similar test condition, *P_u_* is 0.34, 0.82, 1.48, and 2.69 kN. (test condition of *L/D_b_* = 5, *D_s_* = 40 mm, and *D_b_* = 75, 100, 125, and 150 mm).

Bell-shape in belled pile (loose sand) has insignificantly affected comparing dense soil, so the findings of the current study have confirmed the influence of the pile geometry; On the other hand, likewise the estimated results of ANFIS, the experimental output has obviously proved the results (i.e., the influence of the bells angle) because *P_ult_* in belled pile is α = 30, 45, and 60°, *L/D_b_* of 5; *D_s_* = 40 mm; *D_b_* = 125 mm, for loose sand test as 1.46, 1.48, 1.35 kN, and for dense soil test as 3.55, 3.02, 3.25 kN (Figure 9b, Figure 10b, Figure 11b, Figure 12b, Figure 13b and Figure 14b). Testing the pile in different embedment ratio has determined the effect of penetration depth across the pile installation. The pullout load capacity increment in a belled pile (loose and dense sand) with the embedment ratio raising (from 1 to 5) have confirmed the effect of penetration depth across the pile installation observed in ANFIS and measured results.

**Figure 15:**
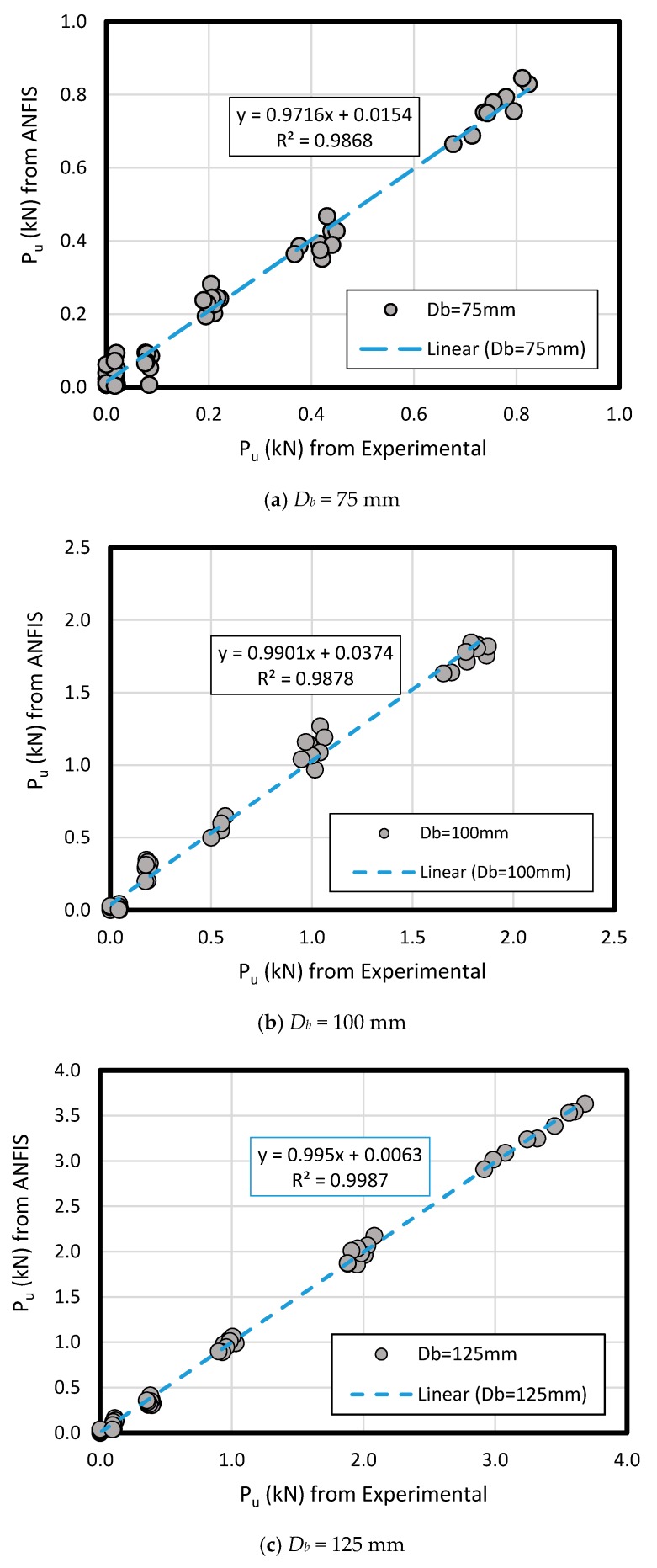

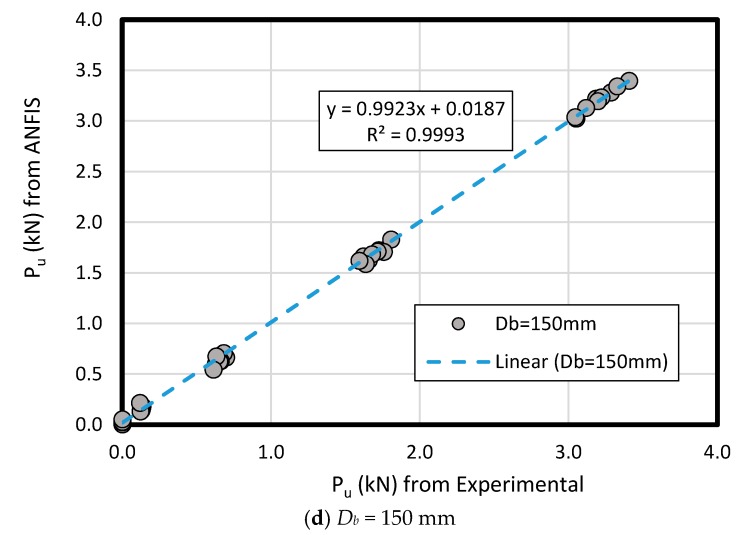
Comparison between measured and ANFIS predicted results of *P_u_* for various *D_b_* in dense sand.

**Figure 16:**
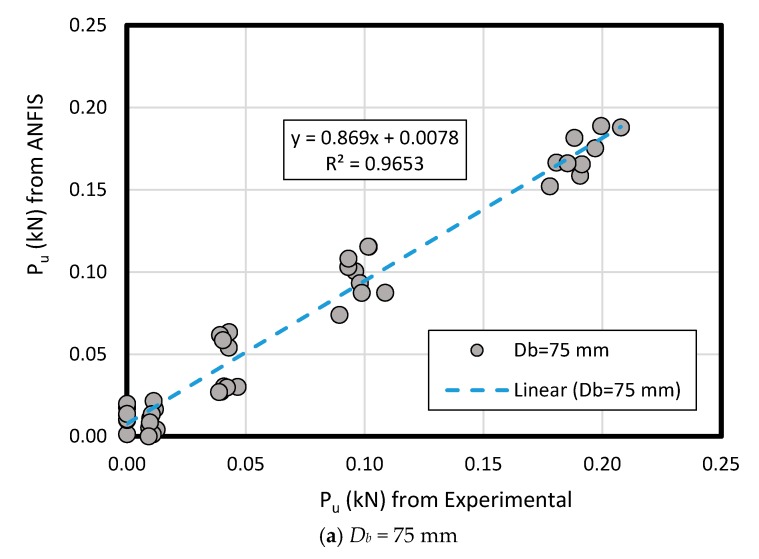

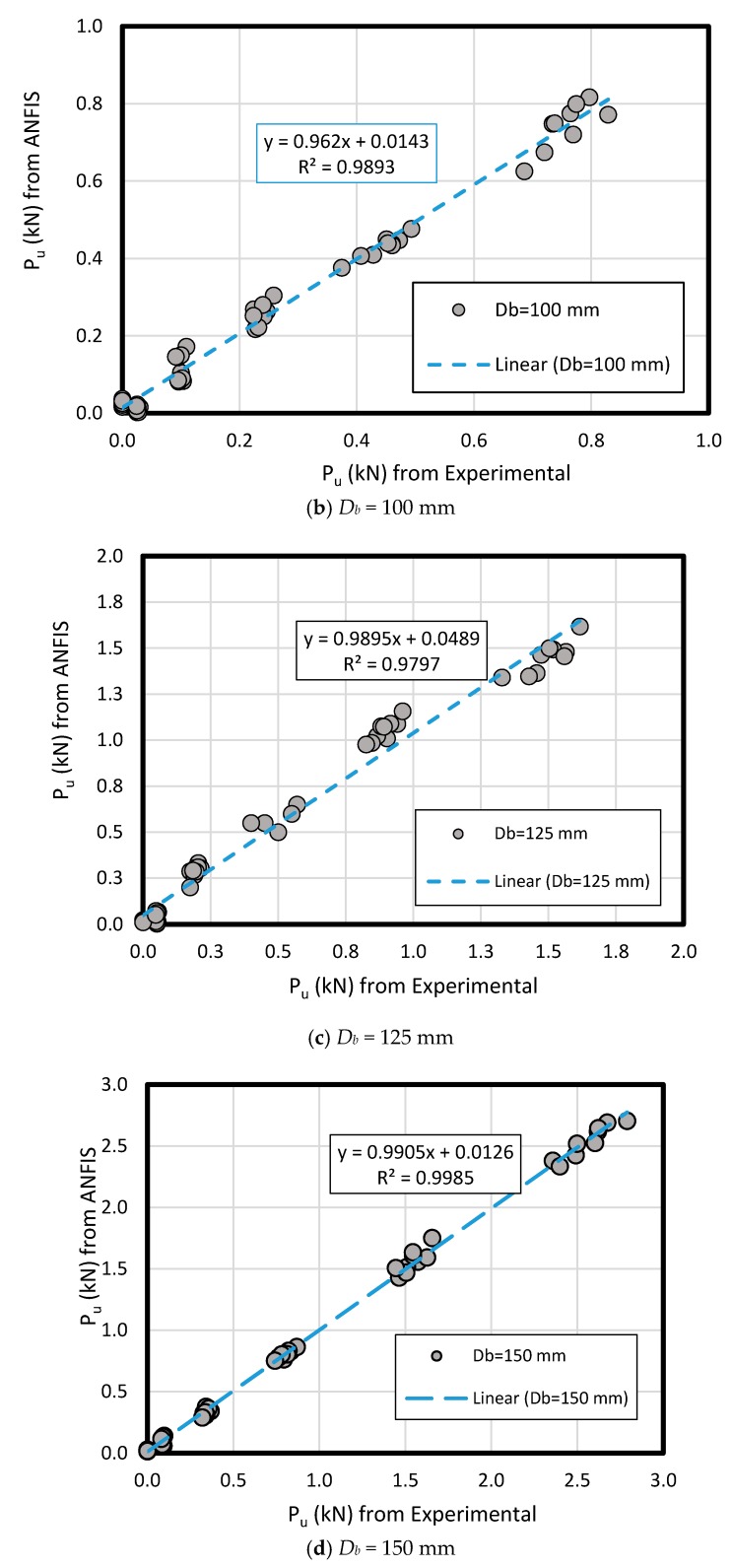
Comparison between measured and ANFIS predicted results of *P_u_* for various *D_b_* in loose sand.

**Figure 17:**
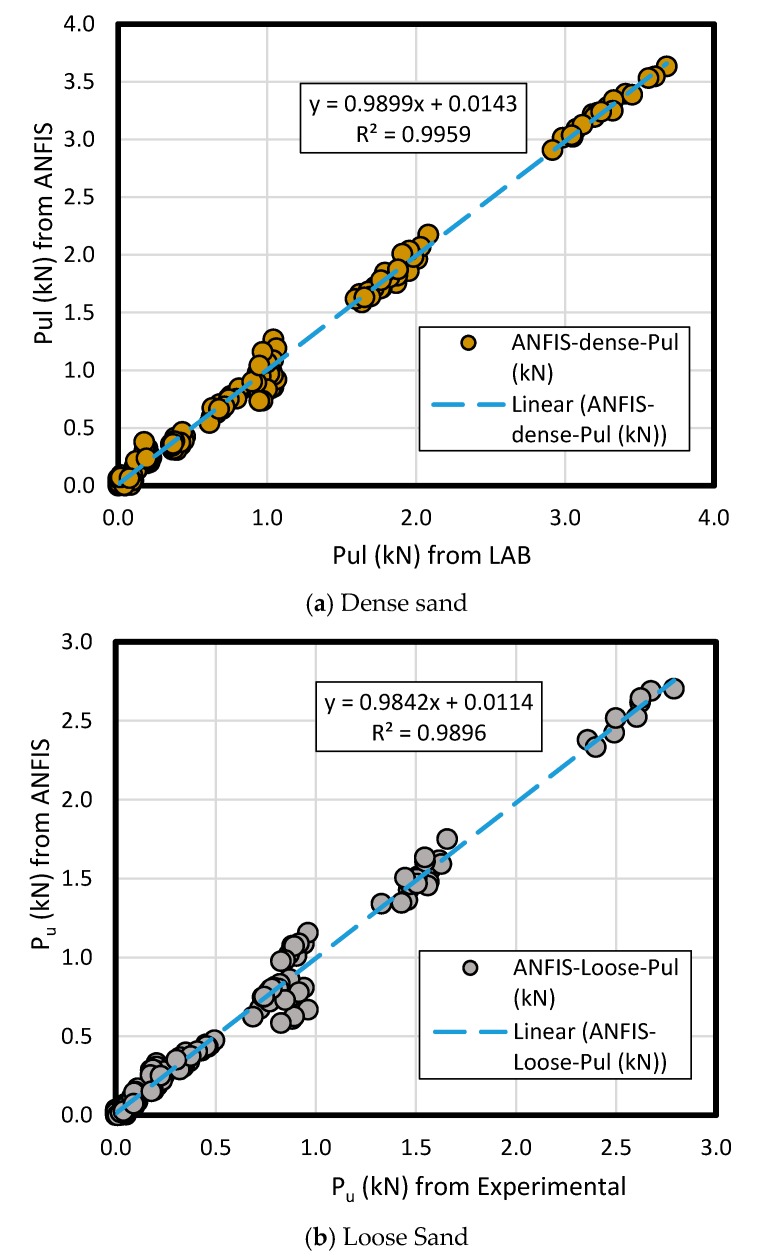
The results of *P_ult_* obtained from ANFIS prediction model and measured from the laboratory; (**a**) dense sand, (**b**) loose sand.

The results of measured data compared to ANFIS prediction results in loose and dense sand have comprised the pullout load (*P_u_*) for predicted and measured method according to the key inputs’ variation like bell’s angle, enlarged base diameter (*D_b_*), and shaft diameter (*D_s_*) along the various embedment ratio (*L/D_b_*)( Figure 15 and Figure 16). As a comparison between the experimental and ANFIS output data in both sand types, the results have followed various diameters (*D_b_* of 75 mm, 100 mm, 125 mm, and 150 mm) for dense sand (Figure 15) and loose sand (Figure 16). A plain regression on the measured and predicted output for two sand types had presented R-square = 0.98 indicating ANFIS accuracy, reliability and flexibility with minimum error in the complex phenomenon and adequate correlation to the experimental data (Figure 17).

## 4. Conclusions

In this paper, several machine learning techniques have been studies to model and predict the influence of the angle of enlarged base, shaft diameter, and base diameter, as well as embedment ratio on the ultimate pullout capacity in under-reamed single concrete piles proving the flexibility, reliability, fast operation and accuracy of these techniques. Likewise, the laboratory data, the predicted pullout capacity from machine learning-based methods have mainly depended on shaft diameter (D_s_), enlarged base diameter (D_b_), density condition (i.e., a geological ground condition such the soil density), and penetration depth (or embedment ration, L/D_b_). Considering the experimental and ANN-based data, it can be seen that the influence of the penetration depth across the pile installation is stronger than other factors. Consequently, based on this study, in a comparison of ANFIS to FFNN, RBNN, and GRNN (i.e., 24, 15, 15, and 6), ANFIS with the total ranking value of 24 has gained the highest rank among the machine learning techniques.

## Figures and Tables

**Table 1:** The details of the ANFIS method.

Type	Sugeno
Inputs/outputs	1/4
No. of input membership functions	67 for each input
No. of output membership functions	67
Input membership function type	Gaussian
Output membership function type	linear
No. of fuzzy rules	67
No. of nonlinear parameters	1336
No. of linear parameters	402
No. of epochs	250

**Table 2:** Physical amounts of the uplift resistance within artificial neural network (ANN) and laboratory work.

Material Parameters	Values	Symbol	Units
Soil			
Dry unit weight	14.7, 18.0	$\gamma_{d}$	kN/m^3^
Relative density	Dense—85%	I_d_	%
	Loose—35%		
**Pile**			
Shaft diameter	30-40-50	D_s_	mm
Base diameter	75-100-125-125	D_b_	mm
Base angle	30-45-60	α	º
Embedment ratio	0-1-2-3-4-5	L/D_b_	--

**Table 3:** Comparing various neural network models and results in pullout capacity forecasting of the enlarged base pile.

Technique	Network Result						Ranking						Total Rank
	TR			TR			TR			TR			
	R^2^	VAF	RMSE	R^2^	VAF	RMSE	R^2^	VAF	RMSE	R^2^	VAF	RMSE	
FFNN	0.957	95.677	2.176	0.951	95.040	2.433	2	2	2	3	3	3	15
RBNN	0.968	96.814	1.608	0.913	91.130	4.032	3	3	3	2	2	2	15
GRNN	0.939	93.884	3.001	0.729	72.745	8.005	1	1	1	1	1	1	6
ANFIS	0.998	97.442	0.058	0.995	96.247	1.252	4	4	4	4	4	4	24
